# 2,8-Dimethyl-10-*p*-tolyl-10*H*-phenoxaphosphine

**DOI:** 10.1107/S1600536809009817

**Published:** 2009-03-25

**Authors:** Thashree Marimuthu, Muhammad D. Bala, Holger B. Friedrich

**Affiliations:** a School of Chemistry, University of KwaZulu-Natal, Westville Campus, Private Bag X54001, Durban 4000, South Africa

## Abstract

The title compound (systematic name: 3,6-dimethyl-10-*p*-tolyl-9-oxa-10-phosphaanthracene), C_21_H_19_OP, is a precursor for the preparation of a bidentate xanthene-based ligand, in which the dihedral angle between the toluene ring and the phenoxaphosphine ring system is 83.26 (3)°. The geometry at the P atom is pyramidal, resulting in a longer C—P bond length as compared to the two ring C—P bonds.

## Related literature

For related structures based on the xanthene backbone, see: Marimuthu *et al.* (2008*a* ▶,*b* ▶,*c* ▶). For a related phenoxaphosphine compound, see: Mann *et al.* (1976 ▶). The title compound was synthesised by a modified literature method (Bronger *et al.*, 2004 ▶). For other structurally related ligands, see: Levy *et al.* (1965 ▶); Seibold *et al.* (2008 ▶); Shau *et al.* (2002 ▶).
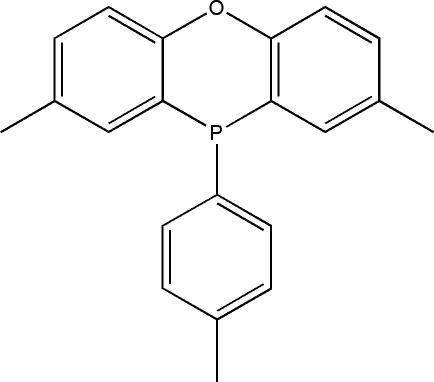


## Experimental

### 

#### Crystal data

C_21_H_19_OP
*M* *_r_* = 318.33
Monoclinic, 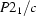

*a* = 10.9363 (3) Å
*b* = 11.6323 (3) Å
*c* = 14.0458 (4) Å
β = 111.532 (1)°
*V* = 1662.13 (8) Å^3^
*Z* = 4
Mo *K*α radiation
μ = 0.17 mm^−1^
*T* = 173 K
0.51 × 0.49 × 0.48 mm

#### Data collection

Bruker APEXII CCD area-detector diffractometer
Absorption correction: none
29900 measured reflections
4013 independent reflections
3507 reflections with *I* > 2σ(*I*)
*R*_int_ = 0.046

#### Refinement

*R*[*F* ^2^ > 2σ(*F* ^2^)] = 0.037
*wR*(*F* ^2^) = 0.108
*S* = 1.07
4013 reflections
211 parameters
H-atom parameters constrained
Δρ_max_ = 0.37 e Å^−3^
Δρ_min_ = −0.27 e Å^−3^

### 

Data collection: *APEX2* (Bruker, 2005 ▶); cell refinement: *SAINT-Plus* (Bruker, 2005 ▶); data reduction: *SAINT-Plus*; program(s) used to solve structure: *SHELXTL* (Sheldrick, 2008 ▶); program(s) used to refine structure: *SHELXL97* (Sheldrick, 2008 ▶); molecular graphics: *SHELXTL*; software used to prepare material for publication: *SHELXTL*.

## Supplementary Material

Crystal structure: contains datablocks global, I. DOI: 10.1107/S1600536809009817/rz2300sup1.cif

Structure factors: contains datablocks I. DOI: 10.1107/S1600536809009817/rz2300Isup2.hkl

Additional supplementary materials:  crystallographic information; 3D view; checkCIF report

## Figures and Tables

**Fig. 1.**
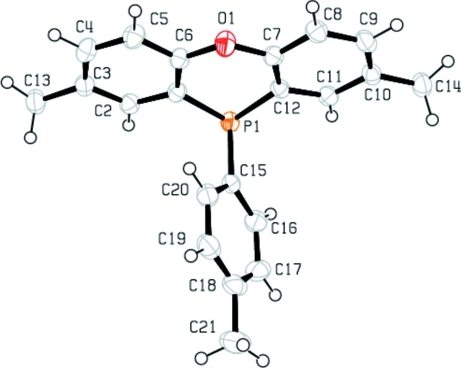
The molecular structure of the title compound. Thermal ellipsoids are shown at the 50% probability level.

## supplementary crystallographic information

### Comment

The title compound was prepared as part of an ongoing study of bidentate and tridentate xanthene-based ligands (Marimuthu *et al.* (2008*a*,*b*,*c*). Similar ligands have shown relative success for the Rh-catalysed hydroformylation of alkenes. The title compound is an example of a modified xanthene backbone where a phosphorous atom has been substituted into the central ring in order to investigate the electronic properties of the final target ligand when complexed to a metal centre. In addition, methyl groups are present on the outer rings in order to increase the solubility of a resulting catalyst. The outer rings of the phenoxaphosphine backbone are nearly coplanar (dihedral angle of 6.56 (2)°). This value is significantly different from the dihedral angle of 15° reported by Mann *et al.*(1976) for 10-phenylphenoxaphosphine, which was observed to have a boat-like conformation about the P—O axis. The C15—P1 bond length for the tolyl group is 1.835 (13) Å, which is longer than the C—P bond lengths of the backbone heterocycle (1.805 (13) and 1.809 (13) Å for C1—P1 and C12—P1, respectively). The longer C15—P1 bond length is due to the pyramidal geometry at the P atom. Hence, the C—P—C angles range from 98.0087 (6) to 101.04 (6)°. The ring of the toluene group is nearly perpendicular to the mean plane through the phenoxaphosphine backbone, forming a dihedral angle of 83.26 (3)°

### Experimental

The synthesis of the title compound was modified from literature (Bronger *et al.* 2004). In an inert nitrogen atmosphere AlCl_3_ (2.5 g, 18.9 mmol) was added to *p*-tolylether (2.5 g, 12.6 mmol) in 9 ml phosphorous trichloride (PCl_3_). The reaction mixture was refluxed for 8 h at 358 K and thereafter the excess PCl_3_ was distilled off at 363 K. At this temperature, excess anhydrous toluene was added to the reaction mixture. The remaining PCl_3_ and toluene was distilled off at 383 K to afford an orange-red residue. The residue was again diluted with 15 ml of toluene and cooled to 273 K, followed by the dropwise addition of 3.6 ml pyridine to the mixture while stirring. After an hour, the resulting salts were filtered off and the yellow residue extracted with toluene. The solvent was removed in vacuo, and the crude product purified by filtration through a short plug of silica gel to yield 2.45 g of the title compound as an oil that solidified at room temperature. X-ray quality crystals were grown from a 2-propanol/dichloromethane (1:1 v/v) solution (Yield: 61%; m.p. 337 – 338 K). Spectroscopic analysis: ^1^H NMR: (400 MHz, C_6_D_6_, δ, p.p.m): 1.98 (s, 6H), 1.90 (s, 3H), 6.78 (d, 2H; J(H,H) = 7.2 Hz,), 6.85 (dd, 2H; CH; J(H,H) = 2.7, 1.7 Hz,), 7.09 (d, 2H; J(H,H) = 8.3 Hz,), 7.30 (dd, 2H; J(H,H) = 1.9, 1.6 Hz,), 7.39 (t, 2H, J(H,H)= 7.9 Hz). MS: m/*z* (%): 357.1 (*M* + K^+^) calculated = 357.1 for C_21_H_19_OPK^+^. FTIR: cm^-1^ = 3009(w), (CH), 2920(s), 1585(w), 1489(m), 1466(vs), 1385(s), 1295(s), 1265(vs), 1231(vs), 909(m).

### Refinement

Non-H atoms were first refined isotropically followed by anisotropic refinement by full matrix least-squares calculations based on *F*^2^ using *SHELXTL*. All hydrogen atoms were first located in a difference Fourier map, then positioned geometrically and allowed to ride on their parent atoms, with C—H = 0.95 - 0.99 Å and with *U*_iso_(H) = 1.2 *U*_eq_(C) for aryl H or 1.5 *U*_eq_(C) for alkyl H atoms.

### Crystal data

**Table:**

C_21_H_19_OP	*F*(000) = 672
*M**_r_* = 318.33	*D*_x_ = 1.272 Mg m^−^^3^
Monoclinic, *P*2_1_/*c*	Mo *K*α radiation, λ = 0.71073 Å
Hall symbol: -P 2ybc	Cell parameters from 8225 reflections
*a* = 10.9363 (3) Å	θ = 2.3–28.4°
*b* = 11.6323 (3) Å	µ = 0.17 mm^−^^1^
*c* = 14.0458 (4) Å	*T* = 173 K
β = 111.532 (1)°	Needle, colourless
*V* = 1662.13 (8) Å^3^	0.51 × 0.49 × 0.48 mm
*Z* = 4	

### Data collection

**Table:**

Bruker APEXII CCD area-detector diffractometer	3507 reflections with *I* > 2σ(*I*)
Radiation source: fine-focus sealed tube	*R*_int_ = 0.046
graphite	θ_max_ = 28.0°, θ_min_ = 2.0°
φ and ω scans	*h* = −14→14
29900 measured reflections	*k* = −15→15
4013 independent reflections	*l* = −18→18

### Refinement

**Table:**

Refinement on *F*^2^	Primary atom site location: structure-invariant direct methods
Least-squares matrix: full	Secondary atom site location: difference Fourier map
*R*[*F*^2^ > 2σ(*F*^2^)] = 0.037	Hydrogen site location: inferred from neighbouring sites
*wR*(*F*^2^) = 0.108	H-atom parameters constrained
*S* = 1.07	*w* = 1/[σ^2^(*F*_o_^2^) + (0.0534*P*)^2^ + 0.586*P*] where *P* = (*F*_o_^2^ + 2*F*_c_^2^)/3
4013 reflections	(Δ/σ)_max_ = 0.040
211 parameters	Δρ_max_ = 0.37 e Å^−^^3^
0 restraints	Δρ_min_ = −0.27 e Å^−^^3^

### Special details

**Table:**

Geometry. All e.s.d.'s (except the e.s.d. in the dihedral angle between two l.s. planes) are estimated using the full covariance matrix. The cell e.s.d.'s are taken into account individually in the estimation of e.s.d.'s in distances, angles and torsion angles; correlations between e.s.d.'s in cell parameters are only used when they are defined by crystal symmetry. An approximate (isotropic) treatment of cell e.s.d.'s is used for estimating e.s.d.'s involving l.s. planes.
Refinement. Refinement of *F*^2^ against ALL reflections. The weighted *R*-factor *wR* and goodness of fit *S* are based on *F*^2^, conventional *R*-factors *R* are based on *F*, with *F* set to zero for negative *F*^2^. The threshold expression of *F*^2^ > σ(*F*^2^) is used only for calculating *R*-factors(gt) *etc*. and is not relevant to the choice of reflections for refinement. *R*-factors based on *F*^2^ are statistically about twice as large as those based on *F*, and *R*- factors based on ALL data will be even larger.

### Fractional atomic coordinates and isotropic or equivalent isotropic displacement parameters (Å^2^)

**Table:**

	*x*	*y*	*z*	*U*_iso_*/*U*_eq_	
C1	0.59638 (12)	1.06591 (11)	0.15703 (10)	0.0274 (3)	
C2	0.53669 (13)	1.05047 (12)	0.22849 (10)	0.0303 (3)	
H2	0.5484	0.9791	0.2636	0.036*	
C3	0.46153 (13)	1.13388 (12)	0.25049 (10)	0.0313 (3)	
C4	0.44183 (14)	1.23579 (12)	0.19518 (11)	0.0361 (3)	
H4	0.3880	1.2940	0.2069	0.043*	
C5	0.49875 (14)	1.25429 (12)	0.12363 (11)	0.0357 (3)	
H5	0.4843	1.3247	0.0868	0.043*	
C6	0.57734 (13)	1.16977 (11)	0.10547 (10)	0.0289 (3)	
C7	0.69900 (12)	1.12246 (11)	−0.00204 (10)	0.0284 (3)	
C8	0.73269 (14)	1.16321 (12)	−0.08200 (10)	0.0340 (3)	
H8	0.7092	1.2391	−0.1071	0.041*	
C9	0.80050 (14)	1.09313 (13)	−0.12506 (10)	0.0347 (3)	
H9	0.8246	1.1222	−0.1789	0.042*	
C10	0.83423 (13)	0.98126 (12)	−0.09135 (10)	0.0310 (3)	
C11	0.79734 (12)	0.94259 (11)	−0.01249 (10)	0.0294 (3)	
H11	0.8177	0.8657	0.0106	0.035*	
C12	0.73184 (12)	1.01137 (11)	0.03436 (9)	0.0264 (2)	
C13	0.40629 (15)	1.11516 (14)	0.33266 (11)	0.0399 (3)	
H13A	0.3553	1.0437	0.3190	0.060*	
H13B	0.3491	1.1798	0.3333	0.060*	
H13C	0.4784	1.1098	0.3993	0.060*	
C14	0.90781 (15)	0.90328 (14)	−0.13659 (11)	0.0387 (3)	
H14A	0.9981	0.8935	−0.0878	0.058*	
H14B	0.9093	0.9371	−0.2001	0.058*	
H14C	0.8641	0.8283	−0.1515	0.058*	
C15	0.85437 (13)	0.98293 (12)	0.24735 (9)	0.0286 (3)	
C16	0.94457 (15)	0.89406 (13)	0.28148 (11)	0.0377 (3)	
H16	0.9249	0.8209	0.2493	0.045*	
C17	1.06328 (16)	0.91088 (17)	0.36216 (12)	0.0474 (4)	
H17	1.1246	0.8494	0.3837	0.057*	
C18	1.09347 (15)	1.01549 (17)	0.41141 (11)	0.0463 (4)	
C19	1.00354 (15)	1.10404 (16)	0.37752 (11)	0.0440 (4)	
H19	1.0230	1.1767	0.4106	0.053*	
C20	0.88574 (14)	1.08866 (13)	0.29636 (11)	0.0358 (3)	
H20	0.8258	1.1509	0.2739	0.043*	
C21	1.22049 (18)	1.0331 (2)	0.50093 (13)	0.0681 (6)	
H21A	1.2587	1.1074	0.4940	0.102*	
H21B	1.2821	0.9713	0.5024	0.102*	
H21C	1.2033	1.0322	0.5647	0.102*	
O1	0.63326 (10)	1.20048 (8)	0.03575 (8)	0.0356 (2)	
P1	0.69921 (3)	0.95166 (3)	0.14160 (2)	0.02660 (11)	

### Atomic displacement parameters (Å^2^)

**Table:**

	*U*^11^	*U*^22^	*U*^33^	*U*^12^	*U*^13^	*U*^23^
C1	0.0250 (6)	0.0274 (6)	0.0299 (6)	−0.0007 (5)	0.0102 (5)	−0.0010 (5)
C2	0.0298 (6)	0.0307 (6)	0.0311 (6)	−0.0013 (5)	0.0122 (5)	0.0003 (5)
C3	0.0279 (6)	0.0352 (7)	0.0316 (6)	−0.0036 (5)	0.0119 (5)	−0.0059 (5)
C4	0.0349 (7)	0.0322 (7)	0.0445 (8)	0.0023 (5)	0.0185 (6)	−0.0054 (6)
C5	0.0383 (7)	0.0273 (6)	0.0439 (8)	0.0038 (5)	0.0179 (6)	0.0021 (6)
C6	0.0287 (6)	0.0280 (6)	0.0313 (6)	−0.0008 (5)	0.0125 (5)	0.0000 (5)
C7	0.0277 (6)	0.0290 (6)	0.0288 (6)	−0.0008 (5)	0.0105 (5)	0.0001 (5)
C8	0.0373 (7)	0.0331 (7)	0.0332 (7)	−0.0006 (5)	0.0147 (6)	0.0059 (5)
C9	0.0370 (7)	0.0418 (8)	0.0279 (6)	−0.0051 (6)	0.0150 (5)	0.0011 (5)
C10	0.0295 (6)	0.0372 (7)	0.0267 (6)	−0.0050 (5)	0.0110 (5)	−0.0063 (5)
C11	0.0308 (6)	0.0289 (6)	0.0279 (6)	−0.0016 (5)	0.0100 (5)	−0.0026 (5)
C12	0.0263 (6)	0.0285 (6)	0.0239 (5)	−0.0034 (5)	0.0084 (5)	−0.0013 (5)
C13	0.0402 (8)	0.0469 (9)	0.0385 (7)	0.0005 (6)	0.0214 (6)	−0.0047 (6)
C14	0.0404 (8)	0.0456 (8)	0.0346 (7)	−0.0017 (6)	0.0190 (6)	−0.0073 (6)
C15	0.0304 (6)	0.0337 (6)	0.0250 (6)	0.0017 (5)	0.0140 (5)	0.0048 (5)
C16	0.0413 (8)	0.0374 (7)	0.0366 (7)	0.0062 (6)	0.0168 (6)	0.0111 (6)
C17	0.0379 (8)	0.0626 (10)	0.0412 (8)	0.0109 (7)	0.0140 (7)	0.0220 (8)
C18	0.0342 (7)	0.0774 (12)	0.0272 (7)	−0.0079 (8)	0.0111 (6)	0.0112 (7)
C19	0.0416 (8)	0.0588 (10)	0.0334 (7)	−0.0115 (7)	0.0159 (6)	−0.0083 (7)
C20	0.0350 (7)	0.0398 (7)	0.0338 (7)	−0.0013 (6)	0.0141 (6)	−0.0030 (6)
C21	0.0400 (9)	0.1219 (19)	0.0356 (8)	−0.0161 (10)	0.0060 (7)	0.0136 (10)
O1	0.0444 (6)	0.0277 (5)	0.0431 (5)	0.0054 (4)	0.0261 (5)	0.0070 (4)
P1	0.02996 (18)	0.02340 (17)	0.02918 (18)	0.00000 (12)	0.01410 (14)	0.00138 (12)

### Geometric parameters (Å, °)

**Table:**

C1—C6	1.3845 (18)	C12—P1	1.8092 (13)
C1—C2	1.3953 (18)	C13—H13A	0.9800
C1—P1	1.8046 (13)	C13—H13B	0.9800
C2—C3	1.3780 (18)	C13—H13C	0.9800
C2—H2	0.9500	C14—H14A	0.9800
C3—C4	1.390 (2)	C14—H14B	0.9800
C3—C13	1.5021 (19)	C14—H14C	0.9800
C4—C5	1.379 (2)	C15—C16	1.3873 (19)
C4—H4	0.9500	C15—C20	1.390 (2)
C5—C6	1.3896 (19)	C15—P1	1.8352 (13)
C5—H5	0.9500	C16—C17	1.389 (2)
C6—O1	1.3784 (15)	C16—H16	0.9500
C7—O1	1.3794 (16)	C17—C18	1.379 (3)
C7—C8	1.3876 (18)	C17—H17	0.9500
C7—C12	1.3876 (18)	C18—C19	1.382 (3)
C8—C9	1.382 (2)	C18—C21	1.507 (2)
C8—H8	0.9500	C19—C20	1.383 (2)
C9—C10	1.388 (2)	C19—H19	0.9500
C9—H9	0.9500	C20—H20	0.9500
C10—C11	1.3858 (18)	C21—H21A	0.9800
C10—C14	1.4999 (19)	C21—H21B	0.9800
C11—C12	1.3901 (18)	C21—H21C	0.9800
C11—H11	0.9500		
			
C6—C1—C2	117.93 (12)	H13A—C13—H13B	109.5
C6—C1—P1	124.10 (10)	C3—C13—H13C	109.5
C2—C1—P1	117.92 (10)	H13A—C13—H13C	109.5
C3—C2—C1	123.08 (12)	H13B—C13—H13C	109.5
C3—C2—H2	118.5	C10—C14—H14A	109.5
C1—C2—H2	118.5	C10—C14—H14B	109.5
C2—C3—C4	117.25 (12)	H14A—C14—H14B	109.5
C2—C3—C13	120.76 (13)	C10—C14—H14C	109.5
C4—C3—C13	121.98 (13)	H14A—C14—H14C	109.5
C5—C4—C3	121.42 (13)	H14B—C14—H14C	109.5
C5—C4—H4	119.3	C16—C15—C20	118.26 (13)
C3—C4—H4	119.3	C16—C15—P1	117.45 (11)
C4—C5—C6	119.89 (13)	C20—C15—P1	124.28 (11)
C4—C5—H5	120.1	C15—C16—C17	120.68 (15)
C6—C5—H5	120.1	C15—C16—H16	119.7
O1—C6—C1	125.24 (12)	C17—C16—H16	119.7
O1—C6—C5	114.37 (12)	C18—C17—C16	120.93 (15)
C1—C6—C5	120.38 (12)	C18—C17—H17	119.5
O1—C7—C8	114.67 (12)	C16—C17—H17	119.5
O1—C7—C12	124.88 (11)	C17—C18—C19	118.41 (14)
C8—C7—C12	120.45 (12)	C17—C18—C21	121.11 (18)
C9—C8—C7	119.82 (13)	C19—C18—C21	120.48 (19)
C9—C8—H8	120.1	C18—C19—C20	121.14 (16)
C7—C8—H8	120.1	C18—C19—H19	119.4
C8—C9—C10	121.47 (12)	C20—C19—H19	119.4
C8—C9—H9	119.3	C19—C20—C15	120.58 (15)
C10—C9—H9	119.3	C19—C20—H20	119.7
C11—C10—C9	117.27 (12)	C15—C20—H20	119.7
C11—C10—C14	120.08 (13)	C18—C21—H21A	109.5
C9—C10—C14	122.64 (12)	C18—C21—H21B	109.5
C10—C11—C12	122.92 (12)	H21A—C21—H21B	109.5
C10—C11—H11	118.5	C18—C21—H21C	109.5
C12—C11—H11	118.5	H21A—C21—H21C	109.5
C7—C12—C11	118.04 (12)	H21B—C21—H21C	109.5
C7—C12—P1	124.12 (10)	C6—O1—C7	122.19 (10)
C11—C12—P1	117.81 (10)	C1—P1—C12	98.00 (6)
C3—C13—H13A	109.5	C1—P1—C15	100.87 (6)
C3—C13—H13B	109.5	C12—P1—C15	101.04 (6)
			
C6—C1—C2—C3	−0.7 (2)	C20—C15—C16—C17	−0.3 (2)
P1—C1—C2—C3	176.68 (10)	P1—C15—C16—C17	−179.07 (11)
C1—C2—C3—C4	2.4 (2)	C15—C16—C17—C18	1.1 (2)
C1—C2—C3—C13	−176.31 (13)	C16—C17—C18—C19	−1.0 (2)
C2—C3—C4—C5	−2.1 (2)	C16—C17—C18—C21	178.23 (14)
C13—C3—C4—C5	176.57 (13)	C17—C18—C19—C20	0.1 (2)
C3—C4—C5—C6	0.2 (2)	C21—C18—C19—C20	−179.10 (14)
C2—C1—C6—O1	177.32 (12)	C18—C19—C20—C15	0.6 (2)
P1—C1—C6—O1	0.09 (19)	C16—C15—C20—C19	−0.5 (2)
C2—C1—C6—C5	−1.29 (19)	P1—C15—C20—C19	178.12 (11)
P1—C1—C6—C5	−178.52 (10)	C1—C6—O1—C7	10.4 (2)
C4—C5—C6—O1	−177.21 (13)	C5—C6—O1—C7	−170.89 (12)
C4—C5—C6—C1	1.5 (2)	C8—C7—O1—C6	171.17 (12)
O1—C7—C8—C9	179.10 (12)	C12—C7—O1—C6	−9.1 (2)
C12—C7—C8—C9	−0.6 (2)	C6—C1—P1—C12	−8.60 (12)
C7—C8—C9—C10	1.1 (2)	C2—C1—P1—C12	174.17 (10)
C8—C9—C10—C11	−0.1 (2)	C6—C1—P1—C15	94.34 (12)
C8—C9—C10—C14	−179.98 (13)	C2—C1—P1—C15	−82.90 (11)
C9—C10—C11—C12	−1.45 (19)	C7—C12—P1—C1	9.72 (12)
C14—C10—C11—C12	178.41 (12)	C11—C12—P1—C1	−172.23 (10)
O1—C7—C12—C11	179.45 (12)	C7—C12—P1—C15	−93.08 (12)
C8—C7—C12—C11	−0.86 (19)	C11—C12—P1—C15	84.97 (11)
O1—C7—C12—P1	−2.50 (19)	C16—C15—P1—C1	159.40 (10)
C8—C7—C12—P1	177.19 (10)	C20—C15—P1—C1	−19.24 (12)
C10—C11—C12—C7	1.94 (19)	C16—C15—P1—C12	−100.12 (11)
C10—C11—C12—P1	−176.23 (10)	C20—C15—P1—C12	81.24 (12)
